# The Search for a Volatile Human Specific Marker in the Decomposition Process

**DOI:** 10.1371/journal.pone.0137341

**Published:** 2015-09-16

**Authors:** E. Rosier, S. Loix, W. Develter, W. Van de Voorde, J. Tytgat, E. Cuypers

**Affiliations:** 1 Department of Pharmaceutical and Pharmacological Sciences, Toxicology and Pharmacology, University of Leuven (KU Leuven), Leuven, Belgium; 2 Imaging & Pathology Department, Division Forensic Biomedical Sciences, University of Leuven (KU Leuven), Leuven, Belgium; CSIR- Indian Institute of Toxicology Research, INDIA

## Abstract

In this study, a validated method using a thermal desorber combined with a gas chromatograph coupled to mass spectrometry was used to identify the volatile organic compounds released during decomposition of 6 human and 26 animal remains in a laboratory environment during a period of 6 months. 452 compounds were identified. Among them a human specific marker was sought using principle component analysis. We found a combination of 8 compounds (ethyl propionate, propyl propionate, propyl butyrate, ethyl pentanoate, pyridine, diethyl disulfide, methyl(methylthio)ethyl disulfide and 3-methylthio-1-propanol) that led to the distinction of human and pig remains from other animal remains. Furthermore, it was possible to separate the pig remains from human remains based on 5 esters (3-methylbutyl pentanoate, 3-methylbutyl 3-methylbutyrate, 3-methylbutyl 2-methylbutyrate, butyl pentanoate and propyl hexanoate). Further research in the field with full bodies has to corroborate these results and search for one or more human specific markers. These markers would allow a more efficiently training of cadaver dogs or portable detection devices could be developed.

## Introduction

During the decomposition of human and animal remains, a wide spectrum of volatile organic compounds (VOCs) is emitted in the environment. The past few years, the research to characterize this ‘smell of death’ has increased and a wide variety of compounds has already been identified: alkanes, alcohols, acids, esters, ketones, aldehydes, cyclic hydrocarbons, aromatic, sulphur- and nitrogen-containing compounds [1–19]. Pig remains are often used as human analogues (Table 1) because of their similarity in hair coverage, weight, fat to muscle ratio, gut fauna and biochemistry [5, 16, 20]. However, the VOC-profiles of human and animal remains were hardly compared, notwithstanding the fact that they could be interesting to find a human specific marker. The following research groups compared human and animal remains. Degreeff et al. reported that phenylethene and methyl benzoate were more specific for human than animal remains [3]. Cablk et al. compared their experimental results of animal remains with literature results of human remains. They found 11 compounds published on human research which they could not detect in their animal study [2]. Vass suggested that carbon tetrachloride, pentane, decane and undecane appeared to be human specific. Additionally, he saw that 2-methylbutanal was always greater than 3-methylbutanal in the animal remains he studied (pig, deer, dog, cat, squirrel and sheep). However, in human remains he noted that this phenomenon was reversed or that both compounds were equal to each other [19]. Clearly, there are still inconsistencies in literature of the human specific compounds and more research has to be done.

**Table 1:** Overview of species used in previous decomposition studies.

Species	References
Human remains	[8, 12, 14, 15, 17–19]
Pig remains	[1, 4–7, 10, 11, 13, 16]
Mice	[9]
Canines, tuna, chicken, beef steak, lamb and pork chops	[3]
Cow, chicken and pig	[2]
Pig, deer, dog, cat, squirrel and sheep	[19]
Rabbits, mice, frogs, chicks, robins and sturgeon	[12]

Ultimately, a variety of forensic disciplines could benefit from these human specific markers. Mainly in the search of human bodies or remains. Thanks to their good olfactory capacity, cadaver dogs are able to locate bodies [17, 21]. At this moment, mostly nonspecific compounds such as cadaverine and putrescine are used to train these dogs. They can find human cadavers with this training, but the use of artificial scents is highly debated. Cadaver dogs trained with these scents did not always react on real cadaver samples (data derived from dog handlers of the Federal Police in Belgium). Training aids appear to be an oversimplification of the decomposition odor [22]. A human specific marker can be used to train cadaver dogs more efficiently and therefore win time to locate a body. Moreover, when a human specific marker is found, it might be possible to develop a portable device that is sensitive enough to locate human remains.

The decomposition can be influenced by many environmental factors such as temperature, humidity, soil type, submersion of the body [23]. Therefore, it is difficult to compare results of research groups that study the decomposing remains outdoors. In this study, we sampled the headspace of 6 human and 26 animal remains that decomposed for 6 months. This study was conducted in glass jars in laboratory environment to pre-concentrate and therefore easily sample the released VOCs. It is also a manner to standardize the methodology with control of the parameters such as temperature and moisture, as much as possible. These samples were collected and analyzed with a validated method using thermal desorber combined with gas chromatography coupled to mass spectrometry (TD-GC/MS) [12]. When the VOC-profiles of human and animal remains were identified, principal component analysis (PCA) was applied on the results to search for (a) human specific marker(s). The aim of our study was to identify VOCs specific for human decomposition.

## Material and Methods

### Setup of experiment

Human and animal remains were allowed to decompose at room temperature in glass jars closed with metal screw caps (1,062 L; Covera Packaging NV, Hoboken, Belgium). In these screw caps, a hole, which was sealed in between sampling, was made to allow headspace sampling. Since the screw caps were not completely air tight, oxygen could enter the glass jar and thus aerobic decomposition was ensured. This study was carried out in laboratory environment to create a concentrated environment and a similar environment between the decomposing remains. In Table 2, the animal species that are used in this study can be found. These animal remains were chosen based on (1) the availability of dead animals and (2) the wide variety of animals useful in this study. For human and pig remains, different organs were removed and stored in the glass jars (Table 3). The human remains were derived from six different bodies. An empty glass jar was placed in the same closet where all the samples were located. The headspace in the empty jar was sampled as a blank once a week together with the other samples.

**Table 2:** Number of samples and time before the remains were put in the jar.

Group of animals	Species and number	Time before decomposition in jar
**Human remains (H)**	6	H a: 7 days in freezer; H b: 1 day; H c: several weeks (probably 19 days); H d: 3 days in freezer; H e: 1 day; H f: 1 day
**Mammals**	9 (1 pig (P), 2 rabbits (R), 3 mice (M), 3 moles (Ml))	P: 1 dayMl: 10, 4 and 4 days; Other mammals: 0 days
**Fish**	1 sturgeon (S)	0 days
**Amphibians**	4 frogs (F)	0 days
**Reptiles**	1 turtle (T)	In freezer for 2 months
**Birds**	11 (2 chicks (C), 3 robins (Ro), 1 song thrush (St), 1 woodpecker (Wo), 1 warbler (Wa), 1 sparrow (Sp), 2 unidentified birds (B))	Ro c: 1 day; Wo: 9 days in freezer; Wa: 5 days in freezer; B a: 5 days in freezer; Other birds: 0 days

**Table 3:** Organs and tissue that were used for human and pig remains. (ND = Not Determined).

Organs/tissue	H a	H b	H c	H d	H e	H f	Hg*	Hh*	Hi*	Hj*	Hk*	P
**Blood**	36,2 g	1,8 g										6 g
**Brain**				33,1 g	35,8 g		ND	ND	ND			
**Fat**		39,3 g	15,8 g		20,2 g	ND	ND	ND	ND	ND	ND	
**Heart**												79 g
**Intestines**		65,8 g		19,7 g	20,4 g	ND	ND	ND		ND	ND	
**Kidney**				25,4 g								
**Liver**	68,1 g	227,2 g	28,4 g	48,9 g	51,8 g	ND	ND	ND	ND	ND	ND	523 g (+ bile)
**Lung**	84,2 g	49,3 g	23,5 g	52,5 g	53,2 g	ND						
**Muscle**	30,2 g	107,5 g	25,4 g		49,7 g	ND	ND	ND	ND	ND	ND	110 g (+ skin + fat)
**Pancreas**				20,7 g								30 g
**Spleen**				24,5 g								
**Stomach**		116,7 g			23,1 g		ND	ND		ND	ND	288 g

* These human remains were only used in PCA’s of human and pig specific compounds.

### Ethics statement

The human remains samples were collected during autopsies. Autopsy samples were directly received from The Institute of Forensic Medicine, University Hospital of Leuven, Belgium (https://www.uzleuven.be/en/forensic-medicine). For our study of human remains, in which no informed consent was necessary because of the donation of anonymous autopsy samples, approval was received from the Medical Ethics Committee of the faculty of Medicine of the University Hospital of Leuven, Belgium. For the animal remains, it was approved by the Ethical Committee of the University of Leuven, Belgium (P121/2013).

### Sampling of VOCs

The air above the samples was drawn through sorbent tubes (prepacked Tenax TA tubes; 200 mg, 89 mm x 6.4 mm o.d.; Camsco, Houston, Texas) for 20 min at a rate of 100 mL/min using an ACTI-VOC pump (Markes, Frankfurt, Germany). After sampling, the sorbent tubes were closed with polytetrafluoroethylene (PTFE) analytical caps. The air was sampled twice a week for the first month; once a week for the next three months and once a month until the samples had decomposed in the jar for 6 months. The sorbent tubes were conditioned prior to sampling for 1 h at 320°C at a flow rate of 100 mL/min helium (Praxair, Schoten, Belgium).

### TD-GC/MS analyses

The VOCs were analyzed using a validated TD-GC/MS method (Turbomatrix 150: Perkin Elmer, Zaventem, Belgium; 6890N-GC and 5975B-MS: Agilent Technologies, Diegem, Belgium) [12]. The TD was used to desorb the VOCs from the sorbent tubes. The sorbent tube was heated to 300°C for 30 min in the primary desorption. The VOCs were transported to a Tenax-coated cold trap, held at 0°C, due to a continuous flow of helium (40 mL/min). The VOCs are pre-concentrated on the cold trap. Secondary desorption was accomplished by heating the trap to 250°C at a temperature rate of 99°C/s and this temperature was maintained for 25 min. The VOCs were injected onto the GC-column (VF-624ms, 60 m x 0.25 mm x 1.4 μm) via a transfer line at 250°C. The temperature of the GC oven was held at 40°C for 1 min, increased to 80°C at 1°C/min, to 120°C at 3°C/min, to 250°C at 5°C/min and maintained this latter temperature for 10 min with a total GC-runtime of 90,33 minutes. Helium was used as carrier gas at a constant pressure of 29 psi. The GC/MS interface was kept at a constant temperature of 280°C. The electron impact ion source was used in positive mode at a temperature of 230°C. The quadrupole mass analyzer was kept at 150°C. Full scan spectra were recorded in a mass range of 15–400 amu. The VOCs were identified using NIST98 mass spectral library. For correct identification, the MS spectra match factor was minimum 70%.

### Statistical analyses

To better understand all the data from the species gathered during six months of sampling and to determine if there were differences between human and animal remains, PCA was conducted by The Unscrambler X (CAMO Software). PCA is a multivariate data analysis that can be used when dimensionality of the data is high and where the possibility of replication is low [7]. The information is projected in principal components (PC). Multiple PC’s can be used. For every PC, every component that is analyzed in the PCA receives a loading. This loading represents how important this component is in a PC to give variation in the scores (in this study, the species). PC-1 contains the greatest source of information, PC-2 gives less information than PC-1, …. Every PC receives a percentage that indicates how much of the variance in the scores is explained using this PC. PC’s can be plotted in a bi- or tri-dimensional system to visualize all the information of a complex dataset. In this study, PC-1 was always plotted with PC-2. In the score-plot, similarities and differences can be detected in the scores, this means for this study: similarities and differences between human and animal remains. The closer the scores are to each other, the more similar they are with respect to the PC’s used. A score-plot has to be interpreted together with a loading-plot. In this loading-plot, the loadings or variables (in this study the VOCs) that are responsible for differences between scores can be found.

The compounds were identified in every sample and collected in spreadsheets. Based on these tables, the frequency of detection for every compound over a period of one, three or six months was calculated as a percentage for every species. The number of compounds for each chemical class (e.g. in 10 samples we saw 3 different alkanes) was expressed as a percentage of the total number of identified compounds. Based on these percentages a PCA-test was performed. The number of observations for each chemical class was also expressed as a percentage of the total observations (e.g. in 10 samples we saw 20 times an alkane). A PCA-test was conducted on these percentages too. Both tests were done after one, three and six months of sampling. After conducting these two PCA’s, the chemical classes (loadings) that were responsible for differences between human and animal remains were further investigated. In this PCA, the VOCs within the chemical class were analyzed using the prevalence of a VOC during the sampling period.

## Results

### General observations

During 6 months, 452 VOCs were found in the headspace of the decomposing remains. Almost every chemical class was represented: alkanes (27), alkenes (28), aromatic compounds (17), cyclic compounds (13), ethers (13), alcohols (44), ketones (55), aldehydes (18), acids (11), esters (66), sulphur-containing compounds (77), nitrogen-containing compounds (71), halogen-containing compounds (7) and others (5). In the blank jar, traces from the laboratory environment (acetic acid, 2-propanone, ethyl ether, dichloromethane, ethanol, ethyl acetate, hexane, chloroform) and from the jar itself (triacetin, 2-ethyl-1-hexanol, 6-methyl-5-hepten-2-one) were found. Also degradation products of the sorbent tube were identified (benzaldehyde, acetophenone, higher aldehydes (octanal, nonanal, decanal)).

**Fig 1:**
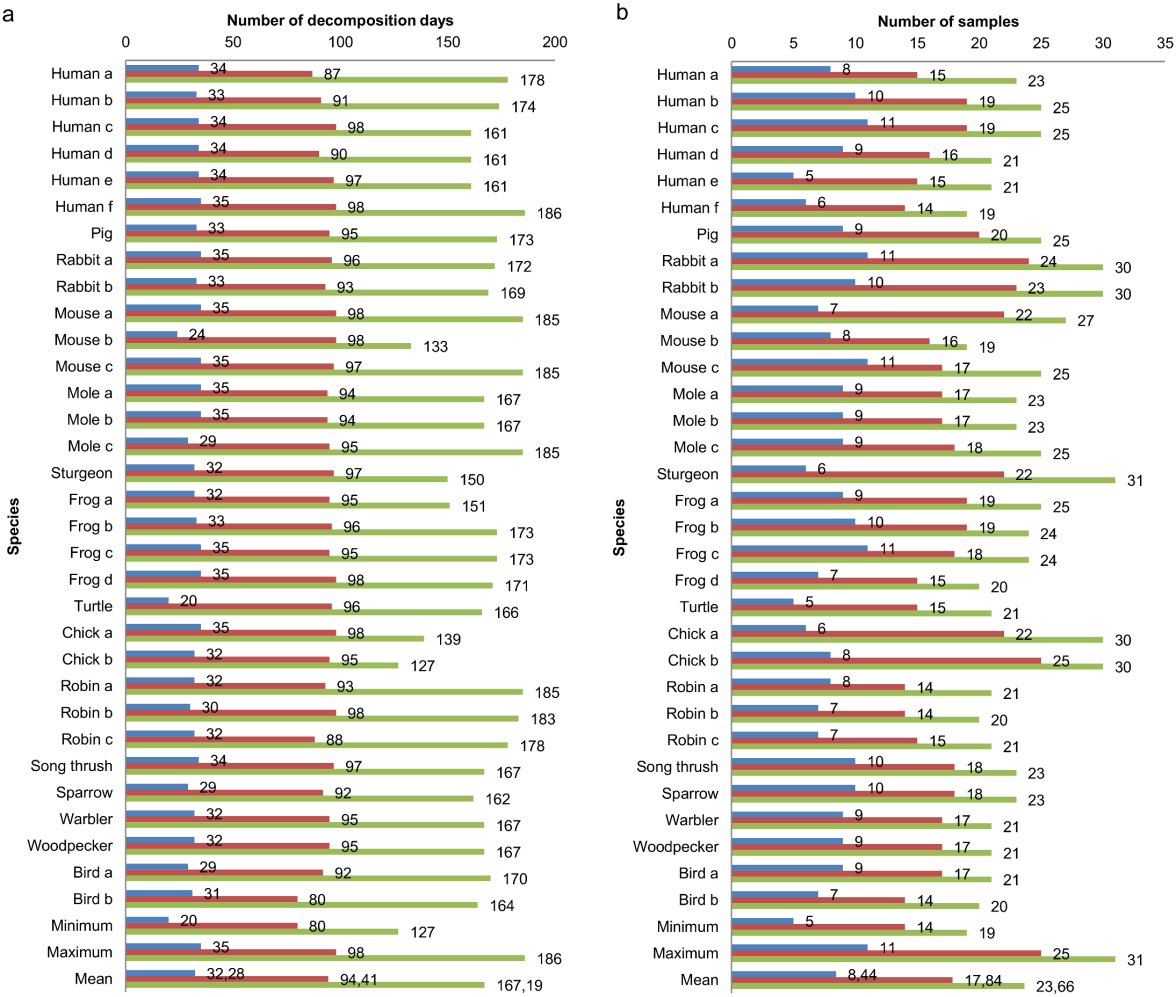
Sample overview. Number of decomposition days (a) and of samples taken (b) of every species after one (blue), three (red) and six months (green) with minimum, maximum and mean of respectively decomposition days (a) and samples taken (b).

In Fig 1, an overview is shown of the number of decomposition days and samples that were taken from every species after one, three and six months. The number of samples varied sometimes because of technical issues whereby samples could not be taken or analyzed. It is important to know that in the first month of sampling, the fresh and bloating stage were ended for every species and they were already in the active or advanced decay.

### Differentiation based on the number of compounds in chemical classes

**Fig 2:**
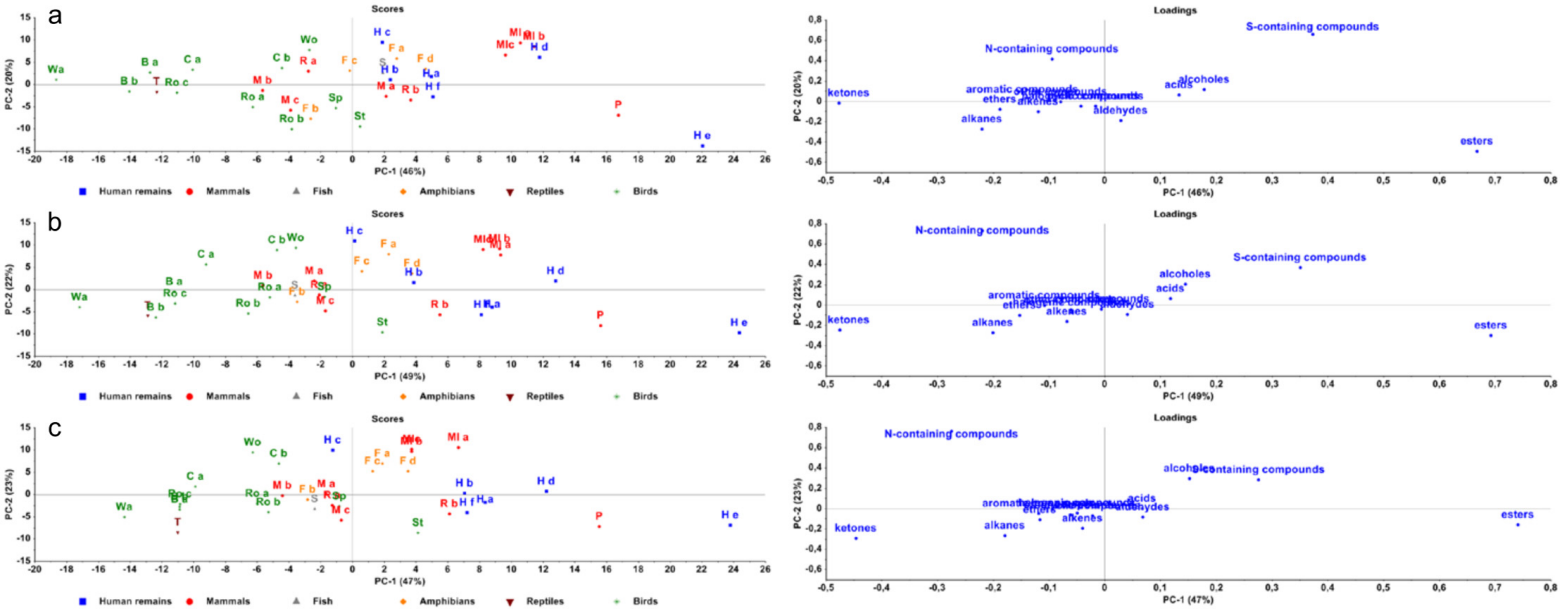
PCA plots of compounds per chemical class. Score- and loadingplots of relative number of compounds in chemical classes after one (a), three (b) and six (c) months.

**Fig 3:**
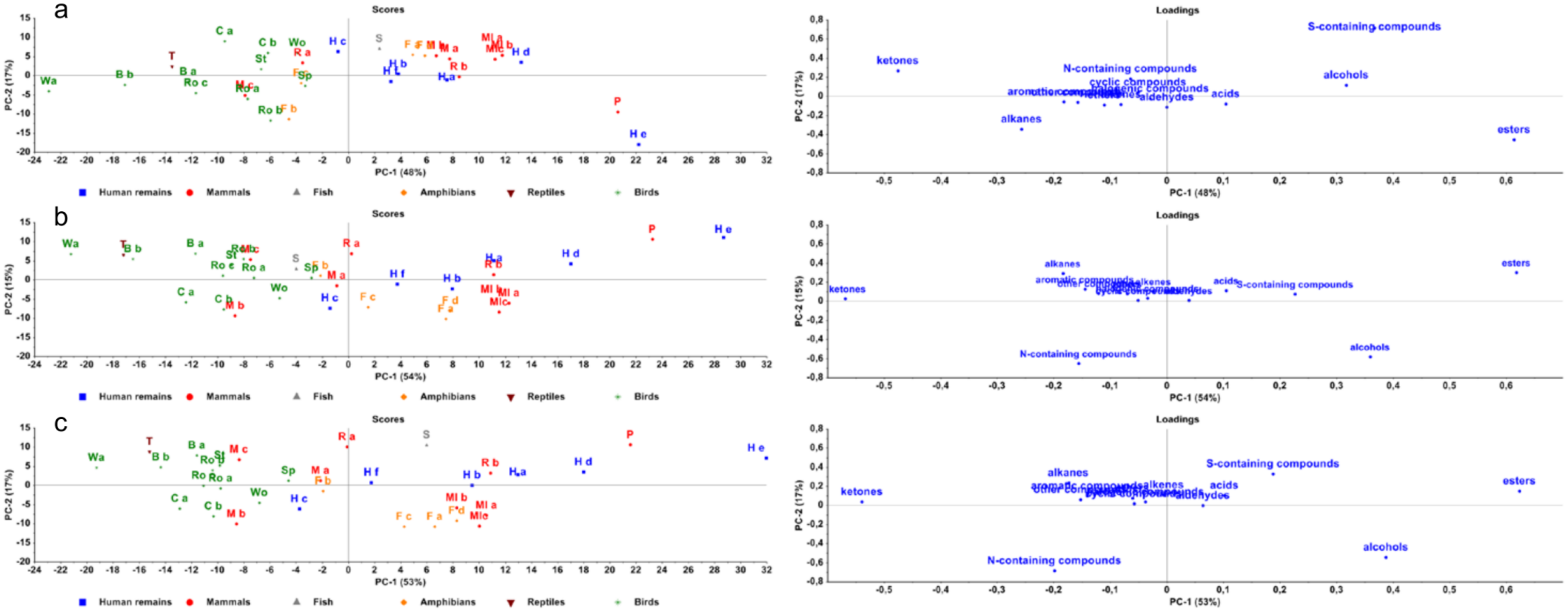
PCA plots of observations per chemical class. Score- and loadingplots of relative number of observations of compounds in chemical classes after one (a), three (b) and six (c) months.

PCA was conducted on the relative number of compounds within a chemical class (Fig 2) and on the relative number of observations of the compounds within a chemical class (Fig 3). With these PCA-results, there could be a more specific search to a human specific compound within the chemical classes of interest.

**Fig 4:**
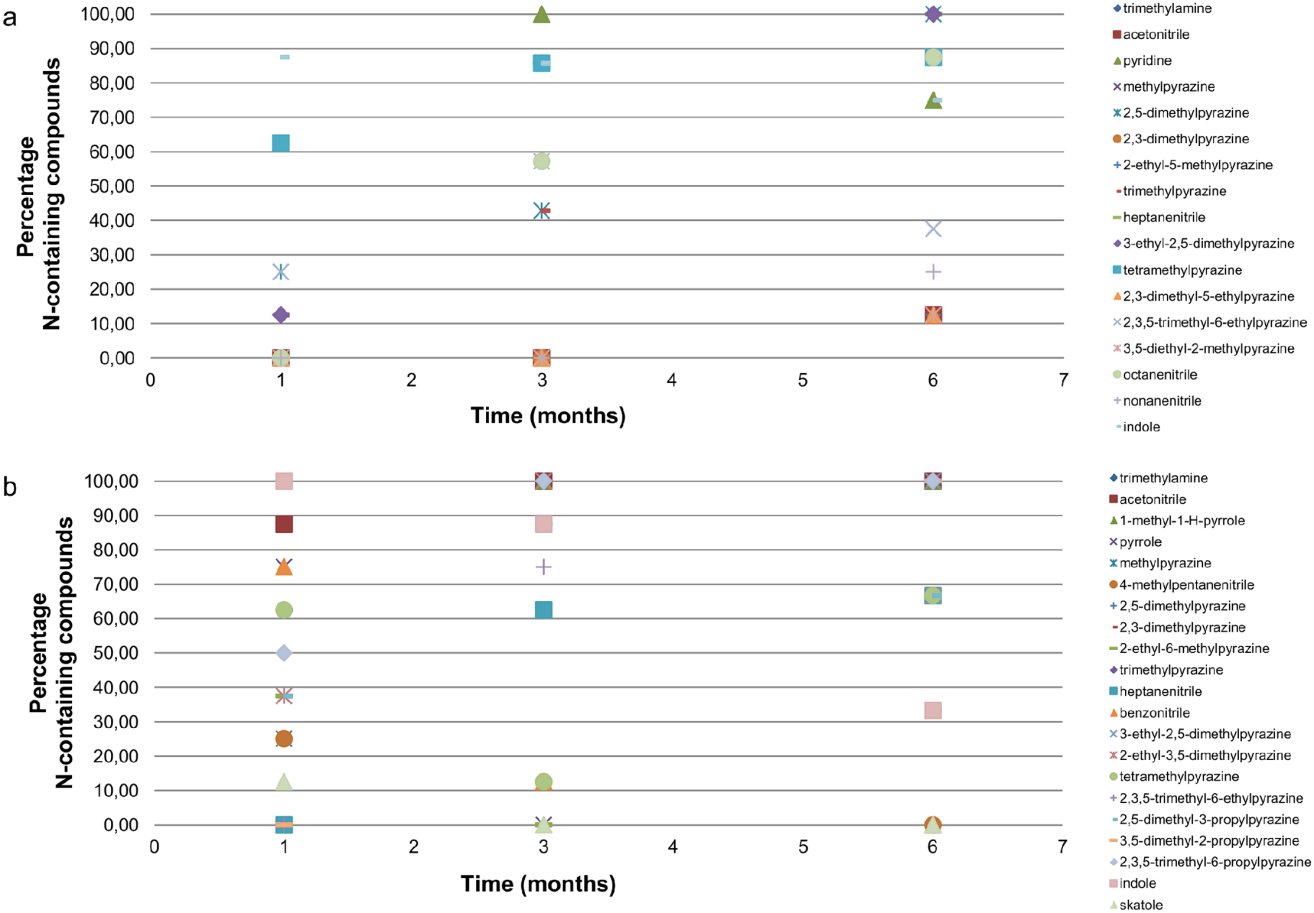
Nitrogen-containing compounds over time. Percentage of nitrogen-containing compounds identified over a time of six months for human remains a (a) and mouse b (b).

After one month, the sulphur-containing compounds are the most important compounds that devide the species in the y-direction in the plot. However, after three months, the sulphur-containing compounds diminished. In Fig 4, the percentage of nitrogen-containing compounds over time is shown for human remains a and mouse b (these two species can be found in a different quadrant in Figs 2 and 3). During the decomposition, the percentage of nitrogen-containing compounds increases. For both species, the loading of the nitrogen-containing compounds in the PCA are similary increasing over time. So this increase is not dependent on the species. When looking at the number of observations of the alcohols, an elevation in loading is observed during time which makes this chemical class interesting. Looking deeper into PC-1 (x-axis), esters and ketones are the chemical classes with the highest loadings and therefore the most important chemical classes that can explain the variance in the score-plot. Therefore, we will go further into these five chemical classes in the search for a human specific compound.

### Interesting VOCs within the chemical classes

#### Sulphur-containing compounds

**Fig 5:**
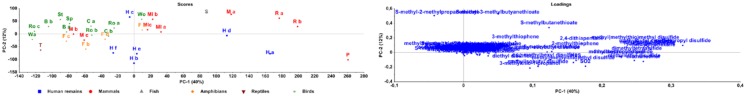
PCA plots of sulphur-containing compounds. Score- and loadingplot of sulphur-containing compounds after six months of decomposition.

A PCA-plot was performed on the sulphur-containing compounds. In Fig 5, the score- and loading-plots are shown after six months of sampling. The human remains are situated in the lower part of the score-plot, with the exception of human remains c. The compounds that are causing this variance are 3-methylthio-1-propanol, methylisobutyl disulfide, SO_2_, methyl(methylthio)ethyl disulfide, diethyl disulfide, CS_2_, methyl hexyl disulfide, H_2_S, methanethiol, methylethyl disulfide, methylisopropyl disulfide and 1-methylthioheptane. None of these compounds are uniquely found in human remains, nor are they found in all the human remains. Hence, this shift of the human samples was probably based on a combination of these compounds. However, there are three compounds that are interesting: (1) 3-methylthio-1-propanol was found in four of the six human samples (not in H c and H f), as well in pig, moles and frogs; (2) methyl(methylthio)ethyl disulfide was detected in five human samples (not in H f) and in pig, rabbits, moles, chicks, woodpecker and unidentified birds; (3) diethyl disulfide was identified in four of six human samples (not in H c and H f) and in the pig remains.

#### Nitrogen-containing compounds

**Fig 6:**
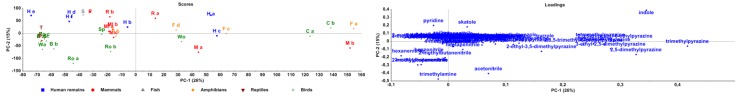
PCA plots of nitrogen-containing compounds. Score- and loadingplot of nitrogen-containing compounds after six months of decomposition.

In Fig 6, the result of the PCA that was conducted on the nitrogen-containing compounds after six months can be seen. Human remains are situated mostly in the upper left quadrant of the score-plot. Human remains a and c are pulled to the other side of the y-axis by pyrazines (2,5-dimethylpyrazine, trimethylpyrazine and tetramethylpyrazine) that were found in high percentages in these two samples. In the loading-plot indole, pyridine and skatole are found in the upper part. Indole was found in most of the species and was therefore not human specific. Skatole was only found in high percentages in sturgeon and occasionally in rabbits, chicks and human remains d. The prevalence of pyridine in human remains ranged from 53 to 96%. Human remains b was the only sample in which no pyridine was identified. This can be explained by the chromatogram: the peak of pyridine was between the overloaded peak of dimethyl disulfide and the overloaded peak of 3-methyl-1-butanol. There is a possibility that pyridine was under those peaks in the chromatogram of human remains b. Pyridine was only seen occasionally in a few other species.

#### Alcohols

In the PCA-plot of the alcohols (S1 Fig), the human remains dwell to the right side of the y-axis together with other species (Ml, P, F and R b). The alcohols with the highest X-loadings are ethanol, 1-propanol, 2-methyl-1-propanol, 1-butanol, 2-methyl-1-butanol and 3-methyl-1-butanol. Those are compounds that were seen in almost every species and therefore not human specific. They have higher loadings because they were more frequently observed in these species.

#### Esters

**Fig 7:**
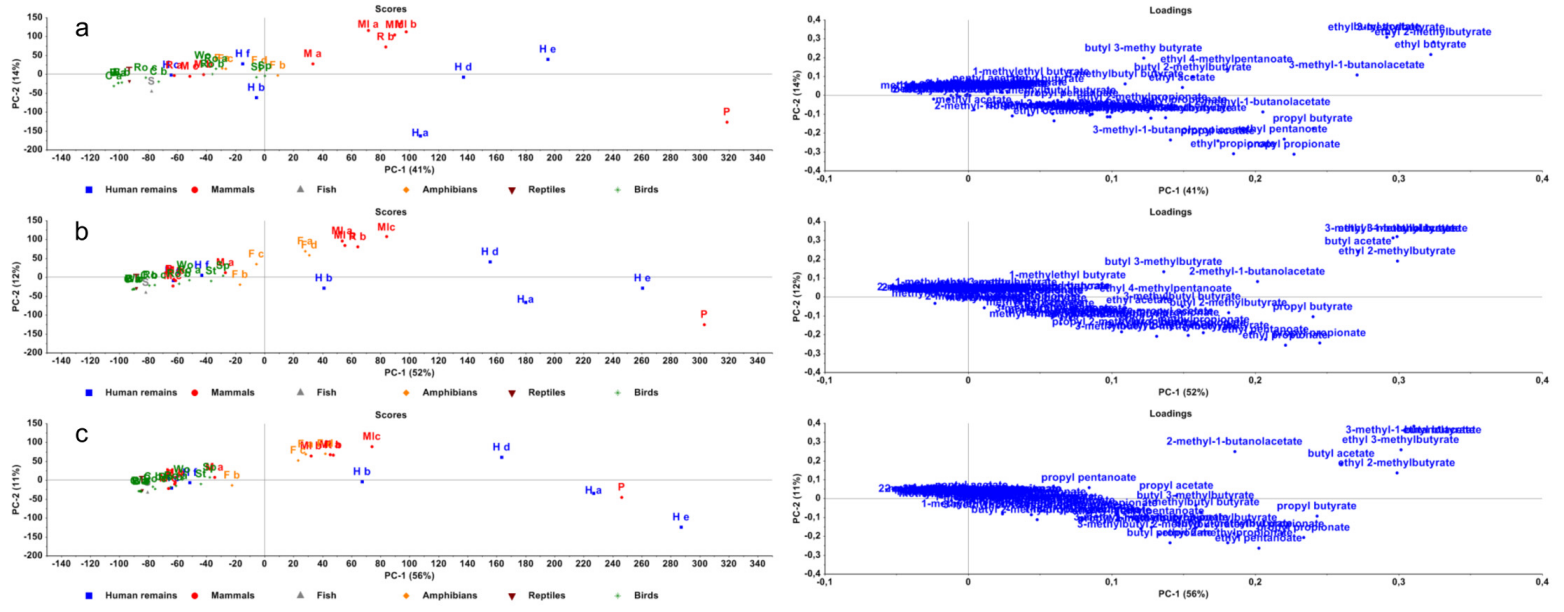
PCA plots of esters. Score- and loadingplots of esters after one (a), three (b) and six (c) months of decomposition.

A PCA was conducted on the esters after one, three and six months (Fig 7). After three and six months, the plot can be divided in two groups. Human and pig remains, moles and frogs are grouped at the right side of the y-axis. Only H c and H f were situated at the left. This separation in two groups is caused by a combination of esters. These esters are not specific for this group, but they had a higher prevalence during the sampling period. Looking at these compounds, the most important esters to search for a human specific marker are ethyl propionate, propyl propionate, propyl butyrate and ethyl pentanoate. These esters were commonly found in human and pig remains, except for H c and H f.

In Fig 7a, the pig remains are completely separated from human remains and other species. This separation was caused by 3-methylbutyl pentanoate, 3-methylbutyl 3-methylbutyrate, 3-methylbutyl 2-methylbutyrate, butyl pentanoate and propyl hexanoate.

#### Ketones

Deduced from the PCA-plot of the ketones (S2 Fig), there is no clear separation of the human remains. They are pulled to the lower part on the score-plot when decomposition is more advanced. This can be explained by a combination of ketones. However, none of these ketones were observed in all human samples neither were they specific for human remains.

## Discussion

Only three research groups suggested human specific compounds previously in literature. In Table 4, a comparison was made between these compounds and the compounds that were found in our study here. Five compounds were not seen in animal remains and therefore still possible human specific markers. However, they were also not seen in human remains in our study. The setup of the study conducted by Degreeff et al. was different from our study since full bodies were used. Also the sampling method was different in the study of Degreeff et al. and Cablk et al. They used solid phase microextraction (SPME) compared to TD. It is possible that due to differences in setup, sampling and analysis method, these compounds were not released or detected in our study [6, 7, 10].

**Table 4:** Suggested human specific compounds found in literature and if they were detected in our study (X) [2, 3, 19].

VOC	References	Detected in human remains in our study	Detected in animal remains in our study
**Phenylethene**	Degreeff et al.	-	-
**Methyl benzoate**	Degreeff et al.	-	X
**Propanoic acid**	Cablk et al.	X	X
**Pentanoic acid**	Cablk et al.	X	X
**Hexanoic acid**	Cablk et al.	-	X
**Butyl butyrate**	Cablk et al.	X	X
**Pentyl hexanoate**	Cablk et al.	-	-
**Hexyl hexanoate**	Cablk et al.	-	-
**2-hexenal**	Cablk et al.	-	-
**2-octen-3-ol**	Cablk et al.	-	-
**Tetrachloroethylene**	Cablk et al.	-	X
**Cyclohexanone**	Cablk et al.	-	X
**2-ethyl-1-hexanol**	Cablk et al.	X	X
**Pentane**	Vass et al.	X	X
**Decane**	Vass et al.	X	X
**Undecane**	Vass et al.	X	X
**3-methylbutanal > 2-methylbutanal**	Vass et al.	3-methylbutanal was detected more frequently than 2-methylbutanal in both human and animal remains	

**Fig 8:**
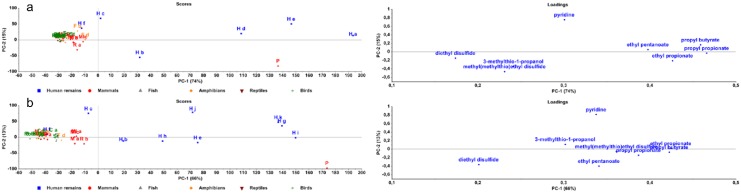
PCA plots of specific markers. Score- and loadingplots of possible human and pig specific markers after six months of decomposition (a) and without frozen remains and with extra samples after one month (b).

In our study, it was not possible to separate the human remains from all the animal remains. However, a combination of eight VOCs is suggested to be human and pig specific: diethyl disulfide, methyl(methylthio)ethyl disulfide, 3-methylthio-1-propanol, pyridine, ethyl propionate, propyl propionate, propyl butyrate and ethyl pentanoate. When a PCA was conducted using only these eight compounds, the score-plot could be divided in two groups with on the right side the human and pig remains, with the exception of human remains f (Fig 8a). It must be noted that different remains were frozen before decomposition was started. Bacteria species may change since bacteria can be knocked down during freezing. Therefore, VOCs released during the decomposition can differ. For that reason, PCA-plot was done over without the frozen samples (H a, H d, T, Wo, Wa and B a) with five extra human samples from a follow-up experiment (H g-k) (Fig 8b). Already after one month of decomposition, separation of human and pig remains could be seen (with the exception of H f) which corroborate previous results.

3-methylthio-1-propanol can originate from methionine via deamination and decarboxylation by yeasts [24, 25]. The two sulfides, diethyl disulfide and methyl(methylthio)ethyl disulfide can be formed during the degradation of methionine and cysteine, the sulphur-containing amino acids [4, 9, 16, 26]. Also other well-documented sulfides were detected in high concentrations in this study (dimethyl disulfide, dimethyl sulfide). However, the latterly mentioned sulfides were not unique for human remains since they were seen in all animal remains in this study as well as in previous decomposition studies [2–9, 12, 14–19]. Notice that in 2 human remains (H c and H f), these human and pig specific sulphur-containing compounds (3-methylthio-1-propanol, diethyl disulfide and methyl(methylthio)ethyl disulfide) were not detected. This might be due to the fact that these compounds were only found in very small trace amounts. It could be possible that the smaller amount of muscles in H c and H f, and therefore smaller amount of sulphur-containing amino acids, is responsible for the lower number of sulphur-containing compounds (22 and 17 respectively) than the average human sample (27,6). 3-methylthio-1-propanol and diethyl disulfide were not detected in H c. These remains were already decomposing for three weeks before the samples were taken. Since the sulphur-containing compounds are less important further in time, this can explain why H c is lying in the upper part of the score plot, separated from the other human remains. 3-methylthio-1-propanol was previously only detected by Forbes et al. and Kasper et al. in pig and mice remains respectively [6, 9]. Until now, methyl(methylthio)ethyl disulfide and diethyl disulfide were not detected in other decomposition studies.

Nitrogen-containing compounds are released during the decomposition of proteins and nucleic acids [4, 23]. The most discussed nitrogen-containing VOCs of decomposition are cadaverine and putrescine since they are assumed to be key compounds in cadaver dog alerting [17]. Putrescine and cadaverine are released after decarboxylation of arginine and lysine respectively [15, 26]. Both compounds were not detected in this or other decomposition studies. Probably because of their low volatility or because they are quickly metabolized by bacteria after they are released [4, 15, 17]. Dekeirsschieter et al. suggested that 2-piperidone could be a metabolite of cadaverine. They detected this compound in the headspace of all their decaying pig carcasses [4]. Also in this study, 2-piperidone was found, occasionally in the chicks and once in human remains c. Pyridine was frequently found in all human remains but one. It was also not detected in the pig remains. Pyridine is a solvent and reagent that is often used in a laboratory environment. This could explain the occasionally identifications in other species, however not for the human remains samples since blanks and species that were sampled the same day as human remains samples were negative for pyridine. Therefore, it is unlikely to originate from the laboratory environment. Pyridine is not abundant in nature. It was previously detected by Statheropoulos et al. and Dekeirsschieter et al. in human and pig remains (only in forest environment) respectively [4, 16]. Statheropoulos et al. suggested that pyridine was originating from niacin [16], also called vitamin B3. This is a precursor of the coenzymes nicotinamide adenine dinucleotide (NAD) and nicotinamide adenine dinucleotide phosphate (NADP). Niacin can be synthesized in the body from tryptophan or it is found in high concentrations in organ meat, fish, whole grain products and legumes [27].

**Fig 9:**
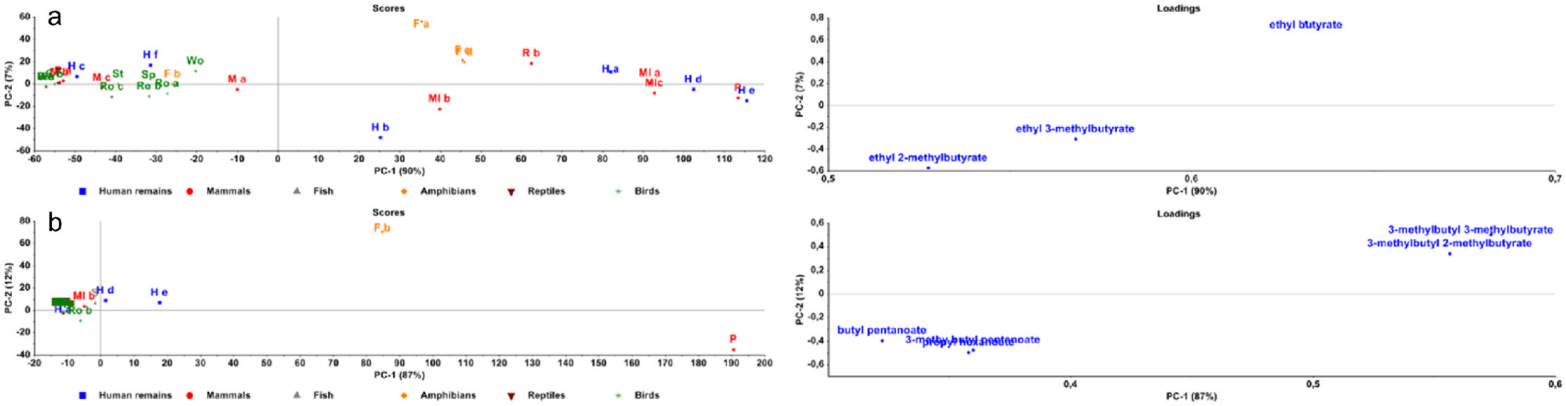
PCA plots of specific markers. Score- and loadingplots of possible omnivore specific compounds after six months (a) and pig specific compounds after one month (b).

Esters are described to be degradation products of muscles, fat tissue and carbohydrates [4, 16]. Kasper et al. suggested that esters are derived from the reaction from acids with alcohols. Since butanoic acid is detected during all the stages and the most prominent acid, they especially found esters from butanoic acid [9]. In this study, a lot of esters were also derived from butanoic acid, but other esters were found as well. Ethyl propionate, propyl propionate, propyl butyrate and ethyl pentanoate, the suggested human and pig specific markers in this study, were also found in other pig studies [6, 7, 10]. Kasper et al. detected propyl butyrate and ethyl pentanoate in decomposing mice, though it was not found in mice in this study [9]. During the analyses of the esters, the combination of ethyl 2-methylbutyrate, ethyl 3-methylbutyrate and ethyl butyrate were interesting, but not specific for human remains. Most of the time, they were detected in frogs, moles, pig and human remains. When these compounds were used in a PCA, two groups could be defined (Fig 9a). In the group at the right of the y-axis, carnivores and omnivores can be found. It is possible that the diet or bacteria in their intestines differ from herbivores and that this has an influence on the production of these compounds. Only F b, H c and H f were at the left side of the y-axis. The number of esters was also lower in human remains c and f (9 and 14 vs. 24 (average)). Probably because of a lower amount of fat tissue (H c) and muscles (H c and H f) that was decomposing in the samples. Some birds (Sp, St, Ro and Wo) are also carnivores or omnivores. They are not situated on the right of the plot, but there is a difference with the other herbivores (R, M and Wa). Those three esters were already found in pig and human decomposition studies [6–8, 10, 14]. Kasper et al. found ethyl 2-methylbutyrate and ethyl butyrate in mice remains, although they were fed with hay food pellets [9].

In all the previous PCA’s, pig and human remains are very similar. This supports the use of pig remains as human analogues [1, 4–7, 10, 11, 13, 16]. However, pig remains could be separated from human remains based on five esters (3-methylbutyl pentanoate, 3-methylbutyl 3-methylbutyrate, 3-methylbutyl 2-methylbutyrate, butyl pentanoate and propyl hexanoate) (Fig 9b). To the best of our knowledge, only butyl pentanoate was identified previously in literature, by Dekeirsschieter et al. in pig remains [4]. This separation is based on one sample of pig remains, which could be not representative. Therefore, this aspect will be investigated further to see if human and pig remains can be separated and a human specific marker can be found.

It should be pointed out however, that there are limitations in our study. Because of the use of glass jars only parts of organs and tissues of humans and pigs could be decomposed. It remains uncertain if other VOCs are released when full bodies are used. However, different tissue (fat tissue, muscle and liver) was sampled, whenever it was possible, to replicate the full body, intestines were added to have the main bacteria that start the decomposition. Using this experimental setup, VOCs could be concentrated in the jars that allowed easy sampling. Another limiting factor of this setup was the absence of environmental parameters that can influence the decomposition and therefore the VOCs that are released. Further investigation in the field with full bodies will have to confirm these results. It should also be noted that using MS identification alone, it is not possible to conclusively identify highly branched esters. However, we could unequivocally identify the human and pig specific compounds when comparing them to the standards (except for methyl(methylthio)ethyl disulfide, 3-methyl butyl pentanoate and 3-methylbutyl 2-methylbutyrate; for these compounds, standards were not commercially available). Notwithstanding its limitations, this study is an important step in the search for human specific markers where VOC-profiles of animal and human remains are identified in a same setup with the identical sampling and analysis method and the profiles could be compared.

## Conclusions

The VOC-profile of 6 decomposing human and 26 decomposing animal remains were identified in a lab-controlled environment. A VOC-profile of 8 compounds (3-methylthio-1-propanol, methyl(methylthio)ethyl disulfide, diethyl disulfide, pyridine, ethyl propionate, propyl propionate, propyl butyrate and ethyl pentanoate) was identified to be specific for human and pigs. However, it is possible that this variation is due to the fact that for both human and pig remains not full bodies were used but only parts of organs and tissues. Additionally, we could separate the pig remains from the human remains based on 5 esters (3-methylbutyl pentanoate, 3-methylbutyl 3-methylbutyrate, 3-methylbutyl 2-methylbutyrate, butyl pentanoate and propyl hexanoate). Despite this separation, more pig remains should be investigated to support these results.

Although the identification of these VOCs is a first step in the search for human specific markers, they were identified in a lab-controlled study. Advantages of this approach are (1) the environmental parameters, such as temperature and moisture, that can be controlled and (2) a concentrated environment that can be generated. Additional research in the field has to corroborate these results in order to see if the environmental parameters influence the release of these compounds and if they are also seen in the VOC-profile of full bodies.

In this study, it was already possible to find compounds specific for human and pigs, further investigation have to search for human specific markers to train cadaver dogs more efficiently and to develop a portable detection device to locate buried human bodies.

## Supporting Information

S1 Fig PCA plots of alcohols. Score- and loadingplots of alcohols after one (a), three (b) and six (c) months of decomposition.
(TIF)

S2 Fig PCA plots of ketones. Score- and loadingplots of ketones after one (a), three (b) and six (c) months of decomposition.
(TIF)
