# Supplementary figures and images for: The Search for a Volatile Human Specific Marker in the Decomposition Process
Source: PLoS One. 2015 Sep 16;10(9):e0137341. doi: 10.1371/journal.pone.0137341 (PMC4572707; doi:10.1371/journal.pone.0137341)

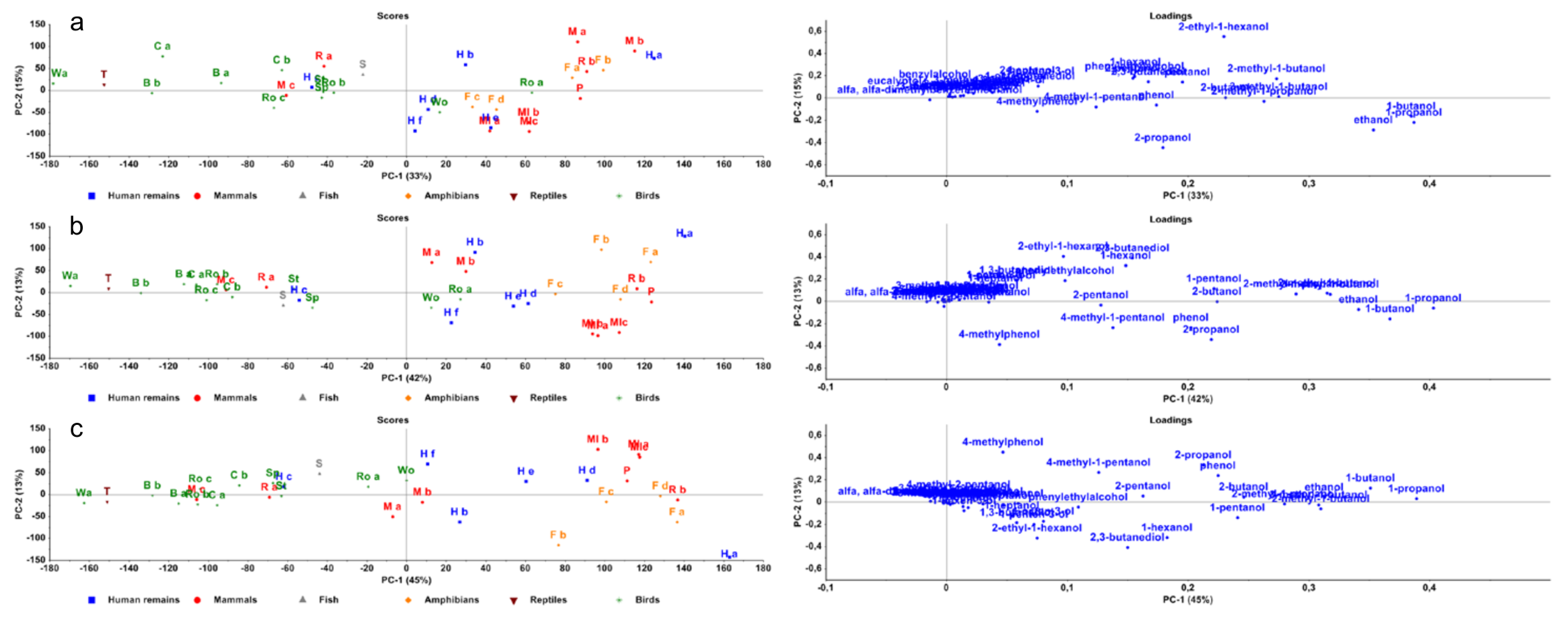

Supplement: S1 Fig — Score- and loadingplots of alcohols after one (a), three (b) and six (c) months of decomposition. (TIF) [file pone.0137341.s001.tif]

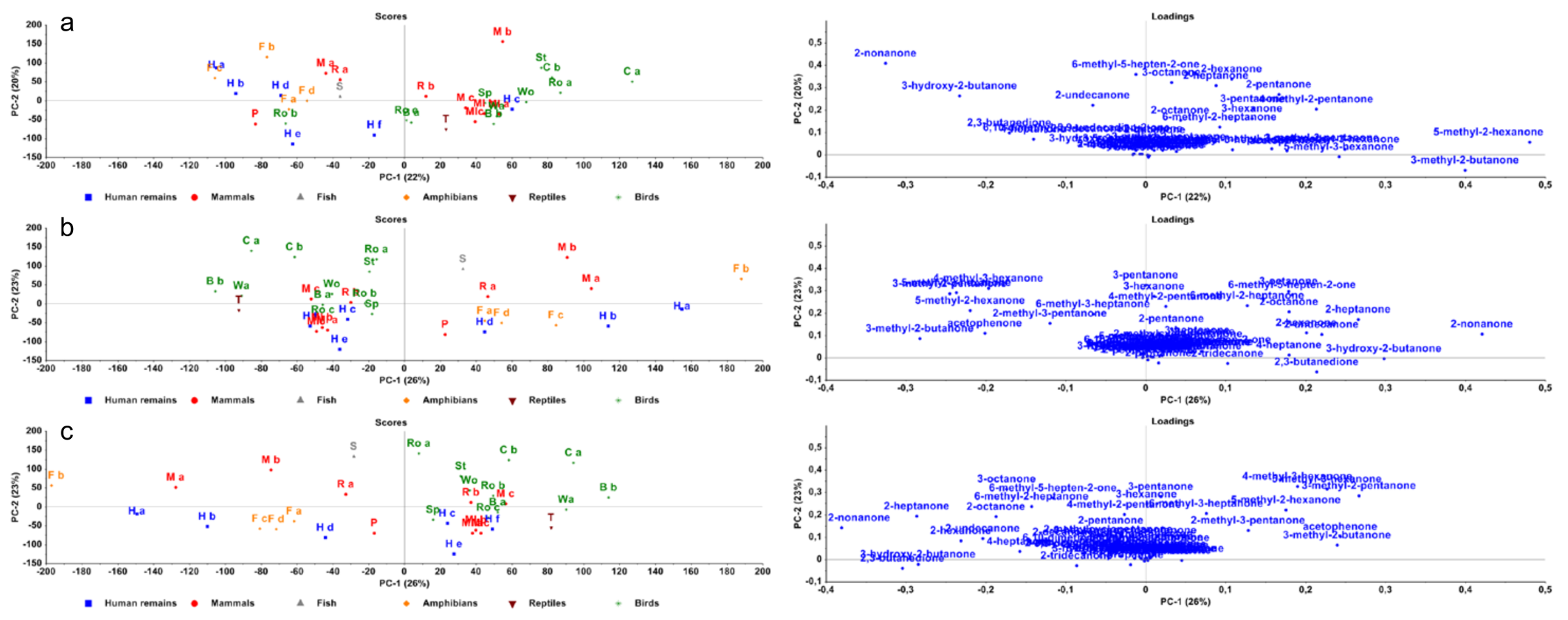

Supplement: S2 Fig — Score- and loadingplots of ketones after one (a), three (b) and six (c) months of decomposition. (TIF) [file pone.0137341.s002.tif]
